# The Influence of Microstructural Arrangement on the Failure Characteristics of 3D-Printed Polymers: Exploring Damage Behaviour in Acrylonitrile Butadiene Styrene

**DOI:** 10.3390/ma17112699

**Published:** 2024-06-03

**Authors:** Sofiane Guessasma, Sofiane Belhabib

**Affiliations:** 1 INRAE, UR1268 Biopolymères Interactions Assemblages, F-44300 Nantes, France; 2 Department of Mechanical Engineering, Nantes Université, CNRS, GEPEA, UMR 6144, F-44000 Nantes, France; sofiane.belhabib@univ-nantes.fr

**Keywords:** crack propagation, additive manufacturing, material extrusion, microstructure, finite element computation, tensile behaviour

## Abstract

This study investigated how printing conditions influence the fracture behaviour of 3D-printed acrylonitrile butadiene styrene (ABS) under tensile loading. Dog-bone-shaped ABS specimens were produced using the fusion filament fabrication technique, with varying printing angles. Tensile tests were conducted on pre-notched specimens with consistent pre-notch lengths but different orientations. Optical and scanning electron microscopies were employed to analyse crack propagation in the pre-notched specimens. In order to support experimental evidence, finite element computation was implemented to study the damage induced by the microstructural rearrangement of the filaments when subject to tensile loading. The findings revealed the simple linear correlation between the failure properties including elongation at break and maximum stress in relation to the printing angle for different pre-notch lengths. A more progressive damage was found to support the ultimate performance of the studied material. This experiment evidence was used to build a damage model of 3D-printed ABS that accounts for the onset, growth, and damage saturation. This damage modelling is able to capture the failure properties as a function of the printing angle using a sigmoid-like damage function and a modulation of the stiffness within the raster. The numerical results demonstrated that damage pattern develops as a result of the filament arrangement and weak adhesion between adjacent filaments and explains the diffuse damage kinetics observed experimentally. This study concludes with a topological law relating the notch size and orientation to the rupture properties of 3D-printed ABS. This study supports the idea of tailoring the microstructural arrangement to control and mitigate the mechanical instabilities that lead to the failure of 3D-printed polymers.

## 1. Introduction 

Additive manufacturing (AM) is among the promising techniques used to design technical parts with a large degree of freedom [1]. One of the main advantages of this technique is the low dependence on the tooling to adapt designs and structures according to the engineering needs [2]. In addition, the local control of the material deposition allows adjusting locally the properties of the materials, which results in developing novel concepts such as gradient-based materials [3], topologically optimised structures [4], metamaterial designs [5], or some combinations of these concepts [6]. These possibilities of material structuring enable several prospects in many research areas such as aeronautics [7], bioengineering [8], civil engineering [9], food [10], and automotive [11]. All processes that use the deposition layer-by-layer framework from digitalised models can be categorised as an additive manufacturing technology. Several techniques fall within this definition, such as stereolithography [12], polyjet [13], selective laser sintering [14] and melting [15], digital light processing [16], electron beam melting [17], laser engineered net shaping [18], and material extrusion [19]. The last process is a popular way to design polymeric materials ranging from single materials such as polylactic acid (PLA) [20] and acrylonitrile butadiene styrene (ABS) [21], to composites with different types of fillers [22]. Several contributions focused on the ultimate properties of ABS under tension. Mogan et al. [23] studied the thermo-mechanical properties of ABS/stainless steel composite using material extrusion. The authors showed that the elongation at break and the ultimate stress of the composites containing up to 10 wt% of steel powder is higher than those of genuine 3D-printed ABS. The comparative study by Golubovic et al. [24] demonstrated that the DLP (digital light processing) route to produce ABS leads to tensile results lower than those from material extrusion. Rodríguez-Reyna et al. [25] conducted a different study, which compared ABS with different feedstock materials such as PLA and nylon/carbon fibres. The authors concluded that the ultimate tensile stress of ABS can be restored by material extrusion with typical values close to 33 MPa. Dusanapudi et al. [26] addressed the relationship between porosity resulting from material extrusion and the efficacy of 3D-printed ABS. By employing a hybrid optimisation technique to fine-tune printing parameters, they achieved a tensile strength of approximately 112 MPa with a porosity level of 24%. He et al. [27] examined the crack behaviour of 3D-printed ABS when subjected to thermomechanical loading. Their research concluded that ABS samples printed with a 0° building orientation exhibited superior fatigue resistance.

The outcome of the extrusion process yields a configuration of filaments varying in cohesion, influenced by both the printing parameters and the inherent properties of the feedstock material [28,29,30]. For instance, several contributions demonstrated the link between the printing temperature and the mechanical performance of ABS. Foltut et al. [31] showed that the brittleness of 3D-printed ABS depends on the printing temperature. Most of the contributions that focus on the link between the thermal behaviour and the mechanical performance of 3D-printed ABS deals with specific fillers such as glass or carbon in an ABS matrix. For instance, Aw et al. [32] studied the tensile and thermal properties of 3D-printed ABS conductive composite. The authors showed a positive correlation between thermal conductivity, tensile strength, and filler weight content. Aravind et al. [33] also studied the thermal and mechanical properties of 3D-printed ABS composite reinforced with glass/carbon fibres. The authors demonstrated the high tensile properties of the ABS composites, namely elongation at break, tensile modulus, and tensile strength, as well as enhanced thermal expansion behaviour.

The extrusion process creates a 2D discontinuity in the space of the part, which results in a particular arrangement of process-induced porosity [34]. This porosity contributes negatively to the mechanical behaviour by generating an amount of loss in stiffness, strength, and elongation at break [35]. In this study, the effect of the process-induced porosity in 3D-printed ABS was explored by allowing crack propagation deviation. ABS serves as an ideal polymer feedstock for validating the experimental and numerical approaches conducted in this study, benefiting from extensive literature and widespread interest in AM [36,37,38]. Compared to PLA, ABS boasts superior properties, such as greater tensile, impact, and flexural strength, enabling its consideration for applications beyond prototyping. This study focused on the correlation between the printing parameters such as the printing angle on the crack deviation processes, which can delay unstable failure of the 3D-printed material. This correlation was studied from both experimental and modelling viewpoints. Through the experimental development, it was shown that a pattern exists between the notch orientation and the printing angle. In addition, the experimental evidence provided a simple quantification of the microstructural arrangement effect on the overall tensile properties such as the elongation at break and the maximum stress. Through the numerical development, the damage law implemented shows that the cracking behaviour can be captured with a small set of parameters, allowing better understanding of the rupture properties in AM materials. 

## 2. Experimental Layout

**Figure 1:**
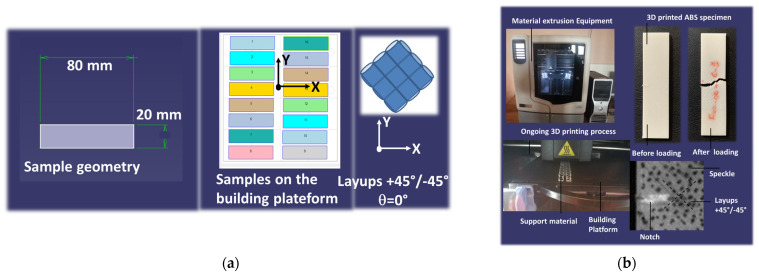
Experimental setup for the 3D printing and testing of ABS: (**a**) ABS specimen geometry and printing configuration; (**b**) ongoing printing process and testing experimental setup.

The feed material used for tensile experiments was the thermoplastic acrylonitrile butadiene styrene (ABS). ABS is the result of the copolymerisation of three monomers: acrylonitrile (32%), butadiene (20%), and styrene (48%). ABS has an average molecular weight of 105,210 g/mol. The ABS grade used in the experiment was P430XL, which was provided by the CADvision company (Guyancourt, France). Regular specimens (length = 80 mm, width = 20 mm, thickness = 2 mm) were used for tensile toughness experiments according to the ISO 13934-1 standard [39] for strip testing. CAD models of the specimens were printed using an uPrint SE 3D printer from Stratasys (Eden Prairie, MN, USA) (Figure 1). 

Samples were printed along the thickness where the plane of construction encompasses the length and width dimensions (Table 1). 

**Figure 2:**
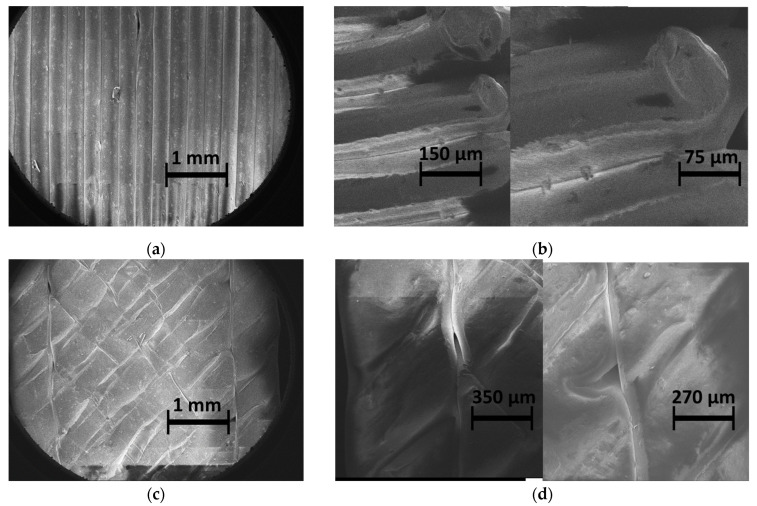
SEM micrographs showing the nature and extent of defects in 3D-printed samples: (**a**) lack of inter-filament adhesion, (**b**) intra-filament porosity, (**c**,**d**) porosity at the junction between the soldered frame and the core filaments.

The as-received ABS filaments (diameter = 1.75 mm) were extruded to filaments having a diameter of 254 µm through the printer nozzle. Printing was performed with the full dense option corresponding to an infill of 100% but with a varied printing angle (θ). A dissolvable support material was used to ease the adhesion between the building platform and the printed specimens. Filaments were arranged in an orthogonal way with respect to the printing angle. This means that the following layups were generated: −45°/+45°, −15°/+75°, and 0°/+90°, and these correspond to printing angles (θ) of 0°, 30°, and 45°. The built-in software (CatalystEX version 2.1) was used for .stl file preparation of collections of filament paths (Figure 2). 

With typically 16 to 21 samples on the printing platform, 44.98 cm^3^ to 55.49 cm^3^ and 20.66 cm^3^ to 28.25 cm^3^ of feedstock and support materials were required. The information about the print volumes serves as a guidance for the determination of the printing cost and environmental footprint of the ABS material. In addition, it helps with measuring the extent of the volume mismatch between the CAD model and the 3D-printed material [40]. The printing duration varied from 242 min. to 389 min depending on the print volume and the filament arrangement. Indeed, this print time was influenced by the part orientation, as the nozzle travel time was different depending on the filament arrangement [41,42]. The experimental campaign required 8 trays, 136 samples, consumption of 427 cm^3^ and 212 cm^3^ of ABS and dissolvable support, and a total printing duration of 33 h. 

Uniaxial tensile testing was performed on printed specimens to determine the crack propagation behaviour according to the strip method (ISO 13934-1). For this purpose, notches were creating after the printing process in which both length (a) and orientation (ϕ) varied (Table 1). The notch orientation was defined as the relative angle between the crack direction and the transverse direction. Notches are used in tensile testing to determine the fracture toughness of materials [43]. Various standards incorporate the notch in tensile tests, including ASTM F 1473 [44]. This standard has been employed to evaluate and compare the resistance to slow crack growth across various polyethylene pipes. Another relevant standard is ISO 16241:2005 [45], which provides guidelines for notch tensile tests on polymeric materials. Additionally, ASTM E602-03 [46] is utilised for determining sharp-notch strength. In this study, five replicates were used for testing each condition. Prior to loading, speckles were added to the surface of the specimens to track the local deformation of the samples upon mechanical solicitation (Figure 1). Loading was performed using a Zwick universal tensile machine (Roell Group, Ulm, Germany) equipped with a 10 kN load cell. The loading rate was adjusted to 10 mm/min and the testing was performed until specimen failure. Monitoring of the sample deformation was conducted using a high-speed camera Phantom V7.3 from Photron company (Tokyo, Japan) under full resolution (800 × 600 pixels) with a rate varying between 100 and 50,000 fps (frames per second). 

Illustrations of the testing process for different configurations, including the effect of the notch length and orientation, are provided as Supplementary Materials.

Additionally, microscopic fractures were examined using a BlueBox apparatus (INRAE, Nantes, France) on post-mortem specimens, alongside high-speed camera recordings. A series of 2D-stitched images were captured at a high resolution, with each image typically containing 1620 × 1220 pixels, where each pixel measures 3.6 µm. Up to 25 images were collected for each condition, constituting a field of view of 8100 × 6100 pixels, equivalent to an acquired area of 29 × 22 mm.

## 3. Modelling Technique

**Figure 3:**
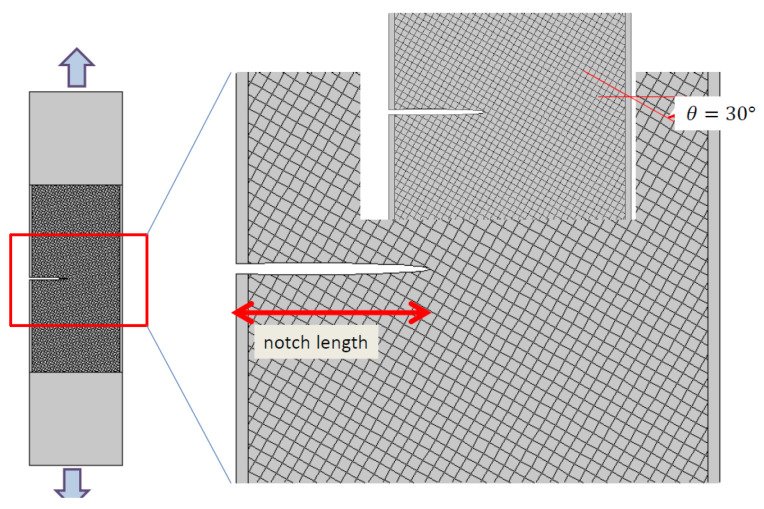
Diagram illustrating the simulated domain utilising the finite element method and the spatial distribution of Young’s modulus based on weak regions between filaments, including the notch (on the **right**), alongside the corresponding finite element meshing (on the **left**).

A finite element (FE) model was constructed to evaluate the progression of damage in printed samples under tensile loading. To numerically examine stress and strain localisation, a basic 2D FE model was suggested. The computations were conducted on slices within the XY plane of the printed samples, as illustrated in Figure 3. 

The overall geometry of the tested specimens was built using primitives under Comsol version 5.2 software (COMSOL France SAS, Grenoble, France). The included geometry represents an array of intersecting filaments resulting from the creation of two consecutive layers. This region consists of a regular pattern of sturdy square cells bounded by weaker interphases. These interphases denote the connections between neighbouring filaments with low cohesion and implicitly capture the impact of the porous network. Consequently, it is appropriate to characterise this stiffness-induced anisotropy using a spatially varying Young’s modulus that adjusts at a scale consistent with the filament’s cross-section. The proposed expression for this is as follows:

(1) E(m(x,y))=EM if p∉∂Γ α×EM×Dεi if p∈∂Γ

where ${\;E}_{M}$ is the matrix modulus representing the stiffness of the as-received ABS ($E_{M}=1.36\;GPa$), α represents the decrease in stiffness due to the lack of cohesion between adjacent filaments, ∂Γ represents the junction region, m(x,y) is sample material point, $\varepsilon_{i}$ is an engineering strain, and D is a damage function that has a sigmoid-like form:

(2) $$D=1-\frac{\beta }{1+e^{-\gamma (\varepsilon_{i}-\varepsilon_{0})}}$$

where:

(3) $$\varepsilon_{i}=\sqrt{{\left(\varepsilon_{11}\left(x,y\right)\right)}^{2}+{\left(\varepsilon_{22}\left(x,y\right)\right)}^{2}+{\left(\varepsilon_{12}\left(x,y\right)\right)}^{2}}\left|\varepsilon_{11}>0;\varepsilon_{22}>0\;\right.$$

where $\beta ,\gamma ,\varepsilon_{0}$ are damage law coefficients, and $\varepsilon_{i}$ is a strain function that accounts for longitudinal, transverse, and shear contributions. 

Equation (3) was proposed by the authors to explain damage behaviour in 3D-printed polymers subjected to severe compression loading [47]. The model examines the progression of damage caused by lateral expansion, resulting in positive strain. It effectively encompasses the primary mechanisms leading to cracking and failure. Validation of the model relied on X-ray microtomography data, affirming the cracking pattern proposed by the model. This model is applicable in scenarios involving tensile loading, where positive strain triggers localised alterations in material stiffness.

Equation (1) adheres to certain constraints imposed by the spatial periodicity of the domain ∂Γ. Specifically, the periodicity of the stiff domains, which are also subject to stiffness degradation, is enforced by the logic of filament crossing. Expression (1) implicitly assumes that the stiff square cells exhibit elastic isotropic material behaviour. Furthermore, the orientation of the domain is adjusted for each printing angle, as depicted in Figure 3. Expression (1) also assumes that the junctions susceptible to damage remain elastic. 

The kinetics of damage is governed by geometrical considerations related to the arrangement of cells and the localisation behaviour at the junctions for samples loaded in the plane of construction.

All computations were carried out using Comsol^®^ software. The solid mechanics module was used to simulate the mechanical behaviour of the 3D-printed ABS under elasticity conditions. The material model selected for ABS assumes isotropic behaviour and stationary conditions are used for all simulations. Regular meshing was applied to the gauge area using square elements, which accounts for the presence of a notch (Figure 3). Quadratic elements were used for the meshing. Each element has two degrees of freedom corresponding to the displacement in X and Y directions. The typical size of the model amounted to approximately 3 × 10^6^ degrees of freedom (dof). Boundary conditions were set in accordance with the experimental testing conditions, involving constrained displacement in the lateral direction and uniform displacement in the longitudinal direction. Geometric nonlinearity was included in the study. The parallel sparse direct solver was used to obtain the predicted stress and strain fields. 

## 4. Results and Discussion

### 4.1. Experimental Results

Figure 2 shows typical SEM micrographs of the microstructure of 3D-printed ABS material. The in-plane observation illustrates the filament alignment along the longitudinal direction (Figure 2a). Within this plane of construction, the necking is represented by the space left between adjacent filaments, which results in a lack of adhesion in the transverse direction. Other defects, such as the reduced section of the filament, also affect the packing of the structure, as shown in Figure 2a. This type of defect can be associated with a varied flow rate and/or change in the nozzle speed. Figure 2b shows porosities within the filament that can be captured from the cross-section views. These porosities are generated by the air trapped inside the filament at the fabrication stage. The intra-filament porosity has a genuine effect on the intrinsic properties of the ABS filament. Figure 2c highlights another type of defect, which is related to the presence of an external frame. This frame contributes to the mechanical stability of the printed structure. However, the junction between the soldered frame and the raster requires an abrupt change in filament trajectory, which generates a triangular porosity (Figure 2d). Similar SEM observations of the microstructure of 3D-printed ABS were obtained by Ahmad et al. [48]. The authors demonstrated that the microstructure of ABS can vary depending on the printing parameters. It may either form a strong, densely packed filament arrangement or exhibit a weaker structure with increased voids, attributed to inadequate adhesion between layers.

**Figure 4:**
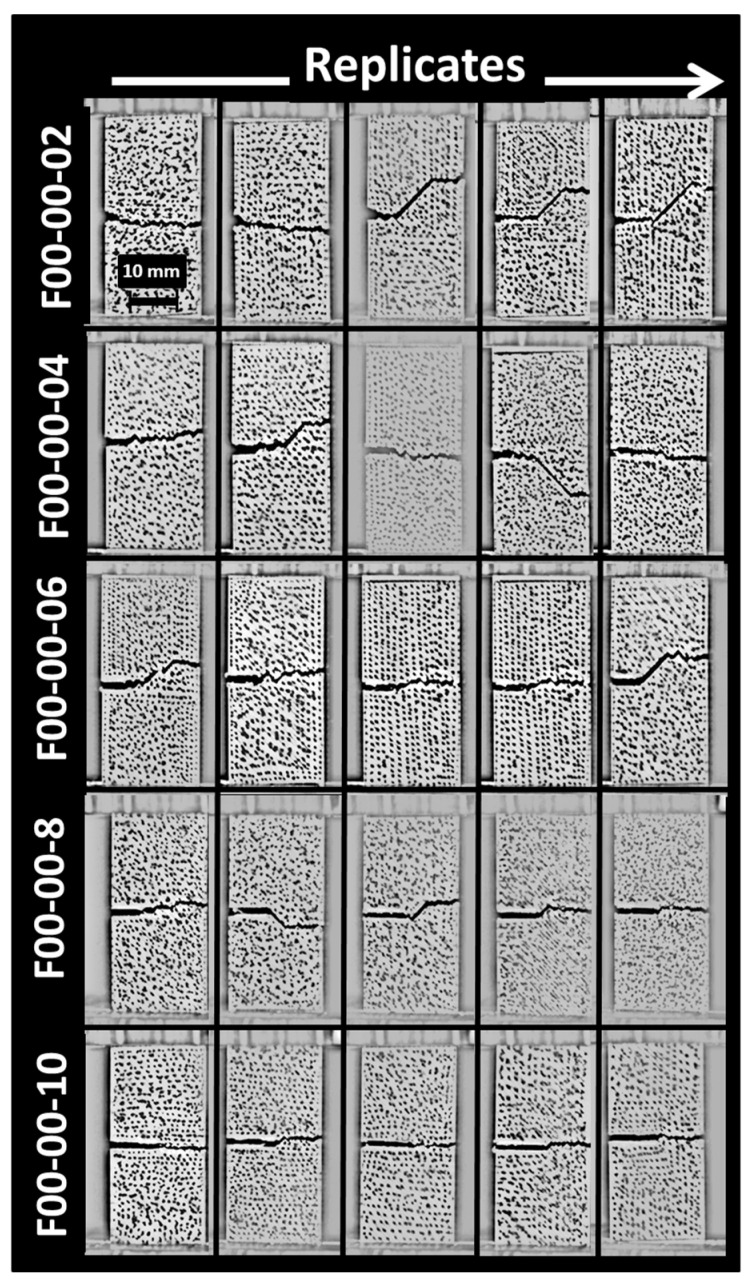
Effect of pre-notch length on crack propagation patterns. Printing angle (θ = 0°) and pre-notch orientation (ϕ = 0°) are fixed.

Figure 4 shows the effect of the pre-notch length on the crack propagation of ABS samples printed according to the printing angle of 0°. 

For these configurations, the generated layups are −45°/+45° and the pre-notch length varies between 2 mm and 10 mm (Table 1). Up to a pre-notch length of 6 mm, crack deviation is witnessed ahead of the crack tip. The deviation matches the filament layups of −45°/+45°, which means that the crack follows more preferentially the junction between the filament where the energy spent for the crack extension is lower compared to a path leading to a fully opening mode. This deviation tends to vanish when the crack length is too large, where a predominant opening mode prevails, especially for 8 mm and 10 mm pre-notch lengths (Figure 4). Dvorak et al. [49] obtained similar results for CT 3D-printed specimens obtained using laser sintering. Crack propagation was found to be dependent on the orientation of the printing layers. 

These experiments yield the crack toughness directly from the standard employed in conducting tensile loading tests on samples with pre-existing notches. Based on these experiments, the critical stress intensity factor was determined using the weight function method for a single-edge crack. As per this approach, the critical crack intensity factor is expressed as:

(4) $$K_{IC}=\sigma \sqrt{\pi \;a}\times f\left(\frac{a}{W}\right)$$

where σ is the maximum stress and a is the notch length.

Equation (4) can be rewritten as:

(5) K1CMPa×m12=Y−×σMPa×π×am

where the geometry factor Y in the typical range of 1.20–2.8 MPa×m12 depends on a and corresponds to the case of an edge crack in a finite-width strip subject to tension loading. The achieved crack toughness of printed ABS is lower than that in a former study by Aourik et al. [50], which suggests the range of 1.70–3.87 MPa×m12.

By excluding large pre-notch lengths (a>8 mm), the measured crack toughness is 2.95 + 0.28 MPa × m^½^. This value corresponds to the experiments conducted with a printing angle of 0° (θ = 0°) and a flat pre-notch normal to the loading direction (ϕ = 0°). 

**Figure 5:**
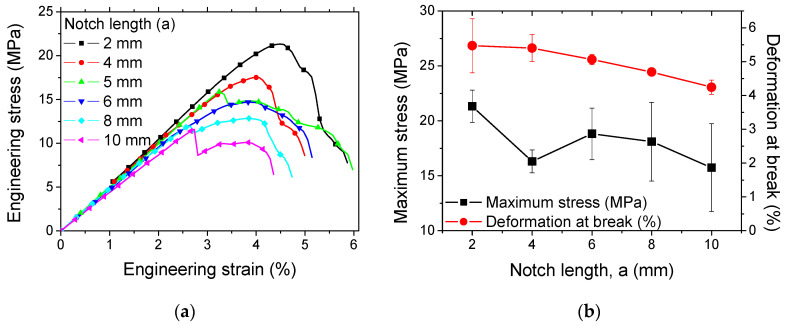
Tensile response of 3D-printed ABS samples, which are pre-notched according to a varied notch length and fixed pre-notch orientation (ϕ = 0°) and printing angle (θ = 0°): (**a**) stress-strain curves, (**b**) ultimate properties as a function of the notch length.

Figure 5a shows the tensile response of the same pre-notched samples for a pre-notch length varying between 2 mm and 10 mm. 

The pre-notch length ratio represents 10% to 50% of the sample width. All samples exhibit nearly the same behaviour, which is a combination of a large elasticity stage followed by a short plasticity stage. The failure of the samples is progressive, which can be quantified by the large difference between the peak stress and the stress at break. The ranking of the materials follows the size of the pre-notches, where the highest responses are attributed to the samples with the smallest pre-notches (Figure 5b).

**Figure 6:**
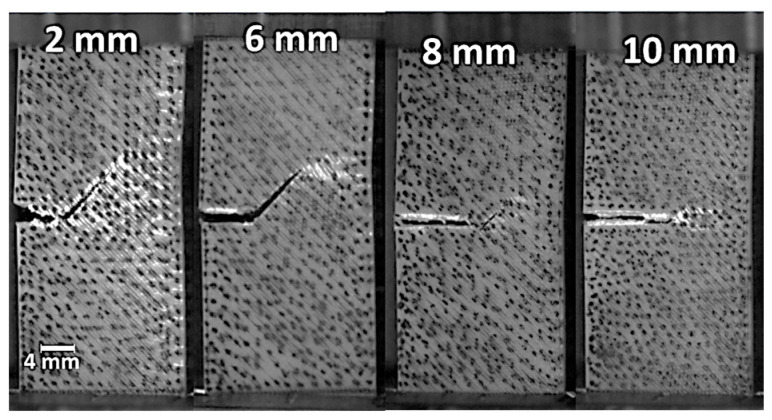
High speed camera recording showing the damage extent for ABS 3D-printed samples with a varying pre-notch length and fixed pre-notch orientation and printing angle.

Figure 6 shows the damage pattern generated from the crack extension of notched samples according to different pre-notch lengths. 

When the pre-notch length is small, there is a high probability of generating a crack deviation at early stages of crack extension. This crack deviation promotes a mixed mode crack propagation, where the shearing effect prevails more when the filament orientation is far away from transverse direction. The printing angle of 0° is a good example of such a situation. As a consequence, a large damaged zone appears ahead of the crack tip, as shown in Figure 6 for a pre-notch of 2 mm. The extent of the damaged zone is reduced when the pre-notch length increases. The extreme case corresponds to a pre-notch of 10 mm where the damage area is reduced to the central line ahead of the crack tip. The results shown in Figure 6 are in agreement with a study by Aourik et al. [50] on the determination of the fracture toughness of 3D-printed ABS material. The authors showed, through experimental evidence and numerical predictions, varying crack and damage patterns according to two different raster angles (namely 0°/90° and −45°/45°). 

**Figure 7:**
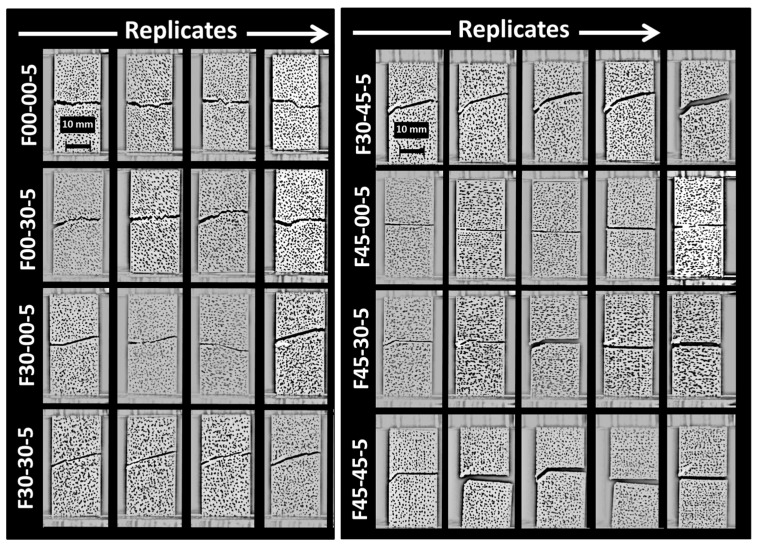
Ruptured 3D-printed ABS samples (FXX-YY-5) imaged using a high-speed camera showing crack propagation patterns as a function of the printing angle (θ = XX) and notch orientation (ϕ = YY) for a pre-notch length of 5 mm.

Figure 7 shows the combined effect of the pre-notch orientation and the printing angle on the cracking behaviour of 3D-printed ABS material. 

These two parameters have a balanced effect on the crack deviation. For the same notch length of 5 mm and the same printing angle of 0°, the change in the notch orientation helps to decrease the deviation of the crack because of the smaller difference between the filament orientation (−45°/+45°) and the crack initial path. This difference starts from 45° and decreases to 0° when the notch angle with respect to the transverse direction increases from 0° (configuration F00-00-5) to 45° (configuration F00-45-5). The same rationale applies for the printing angle of 30° (configurations F30-XX-5). However, the printing angle of 45° does not lead to a significant change in the crack path as the same fully transverse crack propagation occurs irrespective of the initial crack orientation. Marsavina et al. [51] studied different notch geometries in circular bend polymeric specimens obtained by material extrusion. The authors observed similar crack patterns that fit the filament arrangement. However, varied crack deviation was observed depending on the form of the notch. These differences were attributed to contours that were used in the design of the notch. These contours at the crack tip provide a better mechanical resistance against crack propagation by inducing higher plastic deformation. 

**Figure 8:**
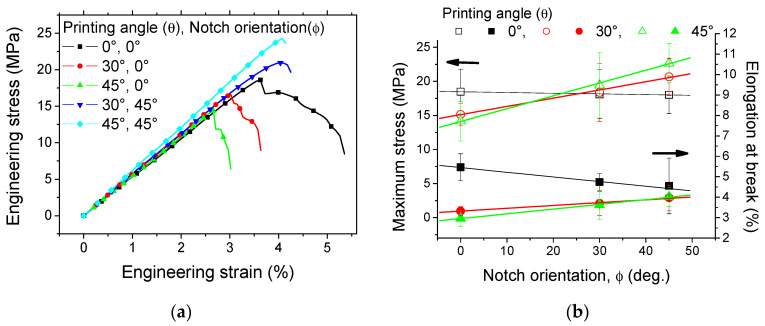
Combined effect of printing angle (θ) and notch orientation (ϕ) on (**a**) tensile behaviour and (**b**) maximum stress and elongation at break.

Figure 8 exhibits the engineering stress–strain response as a function of the printing angle and the notch orientation. 

There is not much variation in the slope of the curves because of the equal length of the notch. However, the rupture properties vary depending on the combination of the two parameters. It was found that the increase in the printing angle decreases both the elongation at break and the ultimate stress, i.e., the stress at the rupture point. In addition, the extent of the progressive damage stage represented by the strain range between the maximum and ultimate stress decreases. This is attributed to the extent of the misorientation between the filament longitudinal direction and the loading direction. The worst-case scenario corresponds to the printing angle of 45°, where only half of the filaments are oriented in the loading direction, while for the other half, the load transfer occurs at the junction between adjacent filaments. For the same printing angle, the change in the notch orientation also affects the elongation at break and the ultimate stress. When the notch angle with respect to the transverse direction increases, Figure 8b shows an improvement in the ultimate properties, namely the stress and the elongation at break. Zolfagharian et al. [52] studied the fracture resistance of U-notched 3D-printed polymers using two 3D-printing routes. The authors demonstrated a correlation between the crack orientation and the failure loads. For samples obtained using material extrusion, the authors observed a higher failure load when the notch was oriented towards the loading direction. This finding is similar to the result shown in Figure 8a. 

In order to further quantify this correlation, the analysis of all printing angles and notch orientations is needed. Figure 8b summarises the effect of both the printing angle and the pre-notch orientation on the maximum stress and the elongation at break. A positive linear correlation between the maximum stress and the pre-notch orientation is observed for two printing angles (i.e., 30° and 45°), whereas the opposite is found for 0°. If the relative difference between the printing angle and the notch angle is taken as a reference, a more inclusive correlation can be found irrespective of the printing angle. This correlation can be fairly represented by a linear function of the form:

(6) $$\sigma_{M}\left(MPa\right)=20.4-0.12\times \left|\gamma \left(deg.\right)\right|$$

where the operator | | refers to the absolute value, $\sigma_{M}$ is the maximum stress, and $\gamma \left(deg.\right)$ = $\theta \left(deg.\right)-$ϕ$\left(deg.\right)$. 

The same trend is also found for the elongation at break, for which the linear function takes the form:

(7) $$\varepsilon_{R}\left(\%\right)=1.71-0.01\times \left|\gamma \left(deg.\right)\right|$$

where $\varepsilon_{R}$ refers to the elongation at break. 

**Figure 9:**
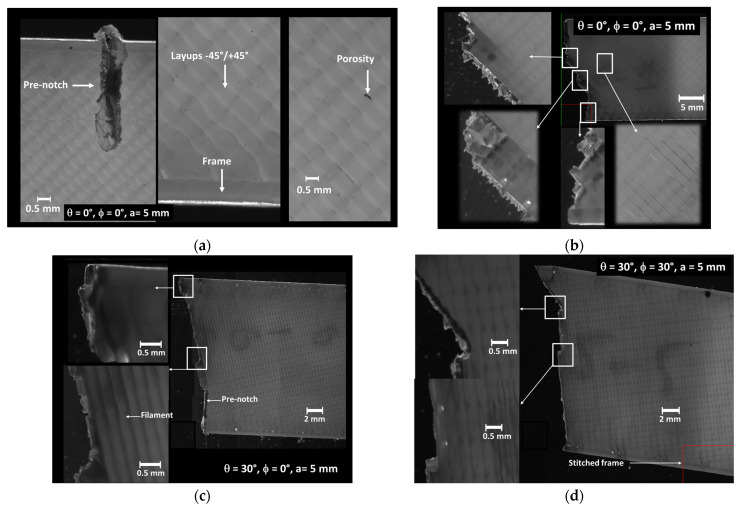
Optical micrographs of the microscopic fracture acquired using the BlueBox apparatus for different printing angles (θ), and pre-notch orientation (ϕ). (**a**) Magnified view of a typical notch θ = 0°, ϕ = 0°, a = 5 mm, and fracture microscopic images for (**b**) θ = 0°, ϕ = 0°, a = 5 mm, (**c**) θ = 0°, ϕ = 15°, a = 5 mm, (**d**) θ = 30, ϕ = 30°, (**b**) ϕ = 15°.

Figure 9a illustrates optical images obtained using the BlueBox apparatus, which provide details of the entire microscopic fractures with the pre-notch length of 5 mm, representing 25% of the sample width. 

Figure 9a shows a magnified view of the notch for a sample printing with layups of −45°/+45° (θ = 0°). The tip of the pre-notch has a rounded shape and both of its lips exhibit a jagged profile due to the machining effect on the raster of the printed sample. It is thus expected that cracking departure from such a notch is affected by an early change in the course. In addition, damage mechanisms are likely to involve a more diffuse stress concentration around the tip and possible varied stress concentration close to the lips. Figure 9b shows the outcome of the printed configuration where the damage pattern resulting from pre-notch opening is found to induce a change in the crack course at least three times prior to the rupture because of the influence of the raster on the crack propagation. A close observation of the crack pattern even shows a larger number of crack deviation attempts, which materialise as the rupture of individual filaments according to a sequence of −45°/+45°. In Figure 9c, the same pre-notch orientation induces a different crack path deviation, which follows the sequence −15°/75°. This deviation fosters a mixed-mode crack propagation, particularly favouring shearing effects when the filament orientation deviates significantly from the transverse direction, exemplified by the 0° printing angle. The magnified views in the same figure highlight two main regions of particular interest: the first one depicts the change in crack path by alternating from −15° to +75° cracking. This alternation is facilitated by the layups where half of the filaments are packed in the +75° direction with respect to the transverse direction. The second region shows a different damage mechanism associated with the external frame, which is more subject to longitudinal extension in the loading direction.

Figure 9d illustrates a fractured pattern for another combination, where the printing angle is maintained at 30° while the pre-notch orientation is corrected to promote a smaller deviation. The fracture pattern demonstrates that the significance of the crack deviation is limited because of the presence of filaments that are slightly misoriented with respect to the initial crack orientation. This difference can be more precisely evaluated from the layups of −15°/+75° induced by the printing angle of 30°. This difference of 15° allows a main crack course in this direction with slight deviations in the direction of 75°. 

### 4.2. Numerical Results

**Figure 10:**
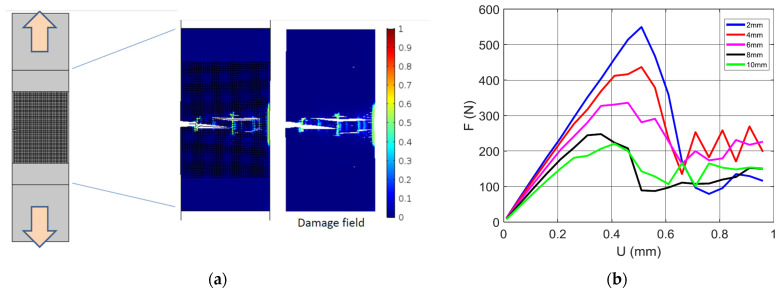
Finite element results depicting the damage induced by the filament arrangement for 3D-printed ABS subject to tensile loading: (**a**) damage growth for a printing angle θ = 45°, (**b**) compilation of predicted force displacement curves for different levels of notch size.

The prediction of the damage evolution for a typical tensile loading of a 3D-printed ABS is depicted in Figure 10a. 

**Figure 11:**
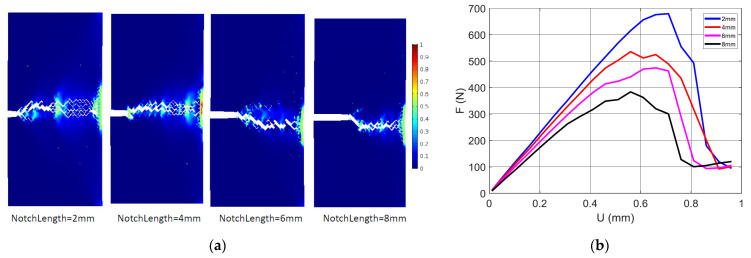
Finite element results depicting (**a**) the damage field corresponding to a printing angle θ = 0°, and for different levels of notch sizes, (**b**) the predicted tensile response for the same notch sizes.

The printing setup corresponds to a printing angle θ = 45°, filament layups of 0°/+90°, and a notch length of 2 mm. The simulation results indicate a multi-damage scenario with several sites ahead of the notch being exposed to a large localisation. The damage follows the orientation of the filament arrangement, which is normal to the loading direction This kind of diffuse damage was observed experimentally, as shown in Figure 6. The damage scenario depicted in Figure 10a is possible because of the weak cohesion between filaments materialised by a junction of low stiffness. Aourik et al. [50] demonstrated, according to finite element computation, that the stress concentration in the vicinity of the notch adheres to the filament arrangement, where the 0°/90° and −45°/45° layups produce different stress distribution heterogeneities, leading to marked bifurcations of the crack path. The effect of the notch size on the predicted load response of 3D-printed ABS is shown in Figure 10b. All configurations were printed with a printing angle θ = 0°. As the loading increases, the notch size negatively affects both the slope and mechanical resistance of the 3D-printed material. In addition, damage accumulation (Equations (2) and (3)) enforces more a progressive decrease in the reaction force, for a load beyond the material tensile strength. The damage is localised for all cases ahead of the notch because of the stress concentration generated by the opening of the notch. When the notch size is large enough, the extent of the damage area, as well as its longitudinal extension, becomes limited. Figure 11a shows the damage field for a printing angle of 0°. In this case, the filament arrangement promotes filaments at 45° from both transverse and longitudinal directions. 

The same trend of a large extent of damage is observed for small notches, such as 2 and 4 mm. In addition, the damage scenario is found to predict a large deviation from the opening mode, but the extension of the damage is limited in the direction normal to the notch direction. At the specimen end facing the notch, the area affected by stress localisation becomes larger. However, due to the localised bending in this area, it is unlikely that the compressive strain would trigger crack departure in the direction opposite to the notch main path. The predicted tensile responses of the same conditions are shown in Figure 11b. The same raking exhibited in Figure 10 is obtained in this case according to the notch size. Additionally, the same progressive smooth decrease in the reaction force is obtained due to the damage growth materialised by the continuous decrease in the material stiffness at the junction points. 

Damage severity in accordance with the observed pore opening mechanism induces more diffuse damage for small notches, as shown in Figure 11a. In the particular case of the printing angle θ = 0°, preferential sites of damage onset are predicted at the corners (largest levels of strain component ε_yy_). Damage growth takes the form of a shearing band (Figure 11a) because of the alternation of positive and negative strains (marked using the levels ε_xx_ = 0 and ε_yy_ = 0). This strain alternation maintains a significant level of mode II propagation along the boundary, which highlights the change in strain sign. 

### 4.3. Validity of the Cracking Behaviour for Other Feedstock Polymers 

In order to check the validity of the cracking scenarios for other polymers used as feedstock materials for material extrusion technology, a comparative study was conducted to determine the role of material properties in the failure induced by the same type of filament arrangement. This study was based on the authors results, which adhere to the same processing and testing conditions. In order to be consistent in the analysis, the same orientations (θ = 0°, layups −45°/+45°) and the same pre-notch characteristics (pre-notch length of 2 mm, pre-notch oriented transversely) were used for all materials. The materials considered were PLA–wood, PLA–hemp, ASA, PLA, PLA-PHA, PETG, nylon, and copolyester. 

**Figure 12:**
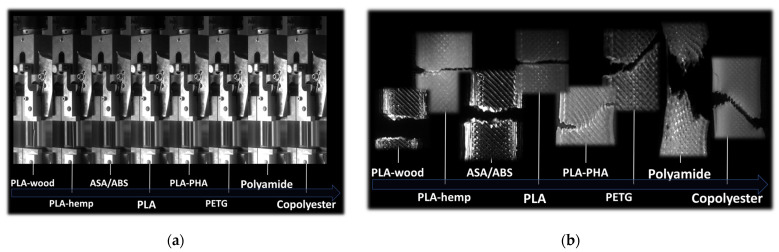
Validity of the cracking behaviour as a function of feedstock materials: (**a**) intrinsic behaviour of as-received polymers; (**b**) typical cracking patterns of a selection of 3D-printed polymers.

Figure 12a compares the elongation at break of the tested materials. Filaments containing fillers such as wood particles and hemp fibres rank among the lowest performing ones due to the lack of compatibility between the PLA matrix and the fillers. The elongation at break is in the range of 6–7% for PLA–wood and PLA–hemp. The material that has the largest elongation at break is copolyester, with an increase of up to 310% in length upon tensile loading prior rupture. Both ABS and ASA lie in the middle range, with a typical elongation at break of about 58%. 

The intrinsic properties of these feedstock materials have a significant influence on the cracking patterns. Indeed, tensile loading performed on notched specimens revealed contrasted trends depending on the ability of the filaments to stretch prior to breakage. Figure 12b shows the evidence of two distinct scenarios of crack propagation for the same collection of polymers. The first is associated with PLA and other polymers sharing a small elongation at break. These materials, when printed using a printing angle of 0°, are supposed to induce a large deviation according to the layups of −45°/+45°. Instead, there is an unstable crack propagation according to a predominant opening mode. Even if the crack path is more or less jagged, the unstable crack does not allow diffuse damage to lead to strong deviation in the crack path. The second collection of polymers starts with PLA-PHA and corresponds to elongation at break as large as 70%. The optical micrographs demonstrate a strong correlation between the raster and the crack deviation. Such deviation is induced by a significant stretching of the filaments in the raster, as in the cases of polyamide and copolyester. 

The comparison between these feedstock polymers shows that ABS polymer belongs to the second collection of polymers thanks to its stretching ability. The crack deviation can be understood as a mechanism of local rupture induced by severe filament stretching between anchoring points. These points are materialised by the crossing of the filaments according to the selected layups. 

## 5. Conclusions

In conclusion, this study found that it is possible to control the cracking behaviour of 3D-printed ABS if the difference between the printing angle and the crack orientation is known. This control was found to be dependent on the filament arrangement, and the crack propagation was explained as an inter-filament cracking process. The crack toughness was found to vary in a large range depending on the crack orientation, namely between 1.20 and 2.8 MPa×m12. The crack deviation results in a large damaged area ahead of the crack tip, indicating a more progressive failure of the material. This study also found that the mechanical stability driven by layups of −45°/+45° is an optimal configuration for uniaxial deformation, but for complex loading in technical parts, adapting the nozzle trajectory to more complex loading paths is needed. ABS polymer is classified within the category of polymers sensitive to the printing angle because of its notable stretchability, which allows crack deflection to occur as a consequence of localised breakage caused by intense filament stretching between anchor points. This opens a new research route for property-driven slicing, where stress state determination from numerical predictions can be used as an input to decide on the optimal approach for part filling. 

## Figures and Tables

**Table 1:** Printing conditions and sample nomenclature. θ: printing angle; ϕ: pre-notch orientation; a: pre-notch length.

Sample	θ (deg.)	ϕ (deg.)	a (mm)
F00-00-02	0	0	2
F00-00-04	0	0	4
F00-00-05	0	0	5
F00-00-06	0	0	6
F00-00-08	0	0	8
F00-00-10	0	0	10
F00-30-05	0	30	5
F00-45-05	0	30	5
F30-00-05	30	0	5
F30-30-05	30	30	5
F30-45-05	30	45	5
F45-00-05	45	0	5
F45-30-05	45	30	5
F45-45-05	45	45	5

## Data Availability

Data are contained within the article and Supplementary Materials.

## Supplementary Materials

The following supporting information can be downloaded at: https://www.mdpi.com/article/10.3390/ma17112699/s1, Video S1: 3D printing—high speed camera recording_1.mp4; Video S2: 3D printing—high speed camera recording_2.
